# Targeting the Epidermal Growth Factor Receptor in Addition to Chemotherapy in Patients with Advanced Pancreatic Cancer: A Systematic Review and Meta-Analysis

**DOI:** 10.3390/ijms18050909

**Published:** 2017-04-26

**Authors:** Jaseela Chiramel, Alison C. Backen, Rille Pihlak, Angela Lamarca, Melissa Frizziero, Noor-ul-Ain Tariq, Richard A. Hubner, Juan W. Valle, Eitan Amir, Mairéad G. McNamara

**Affiliations:** 1 Department of Medical Oncology, The Christie NHS Foundation Trust, Manchester M20 4BX, UK; Jaseela.chiramel@christie.nhs.uk (J.C.); Alison.backen@christie.nhs.uk (A.C.B.); Rille.Pihlak@christie.nhs.uk (R.P.); Angela.Lamarca@christie.nhs.uk (A.L.); Melissa.Frizziero@christie.nhs.uk (M.F.); Noorulain.Tariq@christie.nhs.uk (N.A.T.); Richard.hubner@christie.nhs.uk (R.A.H.); juan.valle@christie.nhs.uk (J.W.V.); 2 Division of Molecular & Clinical Cancer Sciences, University of Manchester, Manchester M20 4BX, UK; 3 Department of Medical Oncology, Princess Margaret Cancer Centre/University of Toronto, 610 University Avenue, Toronto, ON M5G 2M9, Canada; Eitan.Amir@uhn.ca

**Keywords:** advanced pancreatic cancer, epidermal growth factor receptors (EGFR), chemotherapy, rash, *KRAS*

## Abstract

Overexpression of epidermal growth factor receptors (EGFR) occurs in >90% of pancreatic ductal adenocarcinomas (PDACs) and is associated with a poorer prognosis. A systematic review of electronic databases identified studies exploring the addition of EGFR-targeted treatment to chemotherapy in patients with locally advanced (LA)/metastatic PDAC. Efficacy, safety and tolerability of EGFR-targeted therapy were explored using meta-analysis of randomised controlled trials (RCTs). Meta-regression was utilised to explore factors associated with improved prognosis (all studies) and benefit from EGFR-targeted therapy (RCTs). Twenty-eight studies (7 RCTs and 21 cohort studies) comprising 3718 patients were included. The addition of EGFR-targeted treatment to chemotherapy did not improve progression-free (pooled hazard ratio (HR): 0.90, *p* = 0.15) or overall survival (HR: 0.94, *p* = 0.18). EGFR-targeted therapy was associated with increased treatment-related deaths (pooled odds ratio (OR): 5.18, *p* = 0.007), and grade (G)3/4 rash (OR: 4.82, *p* = 0.03). There was a borderline significant increase in G3/4 diarrhoea (OR: 1.75, *p* = 0.06), but no effect on treatment discontinuation without progression (OR: 0.87, *p* = 0.25). Neither G3/4 rash nor diarrhoea were associated with increased survival benefit from EGFR-targeted therapy. The effect of EGFR-targeted therapy on overall survival (OS) appeared greater in studies with a greater proportion of LA rather than metastatic patients (*R* = −0.69, *p* < 0.001). Further studies in unselected patients with advanced PDAC are not warranted. The benefit from EGFR inhibitors may be limited to patient subgroups not yet clearly defined.

## 1. Introduction

Pancreatic cancer is a disease with an extremely poor prognosis (5-year survival of 3%–5%) [1,2,3]. Globally, it is the fourth most common cause of cancer-related death [1,3,4]. Approximately 80% of patients present with locally advanced or metastatic disease [5]. Patients who are diagnosed early and then proceed to surgery have a better chance (7%–25%) of surviving beyond five years after diagnosis [6]. In the European Study Group for Pancreatic Cancer-4 (ESPAC-4) randomised-controlled phase 3 clinical trial, the median survival in patients treated with the adjuvant gemcitabine/capecitabine combination was 28.0 months (95% confidence interval (CI) 23.5–31.5) while in those treated with gemcitabine monotherapy median survival was 25.5 months (95% CI 22.7–27.9) [7].

Single-agent gemcitabine has been the mainstay of treatment for patients with late-stage pancreatic cancer for many years, following a randomised trial of single-agent gemcitabine versus 5-fluorouracil (5-FU), which demonstrated better efficacy for gemcitabine over 5-FU where a clinical benefit response was experienced by 23.8% of patients treated with gemcitabine compared with 4.8% of patients treated with 5-FU (*p* = 0.0022) and median overall survival of 5.65 months versus 4.41 months was reported, *p* = 0.0025) [8]. Gemcitabine is still the treatment of choice for patients with metastatic pancreatic cancer with a borderline Eastern Cooperative Oncology Group performance status (ECOG PS of 1–2).

In 2013, a phase 3 study of albumin-bound paclitaxel (nab-paclitaxel) plus gemcitabine versus gemcitabine monotherapy, in patients with metastatic pancreatic cancer, reported a median progression-free survival of 5.5 months in the nab-paclitaxel-gemcitabine group, as compared with 3.7 months in the gemcitabine group (*p* < 0.001). The median overall survival was 8.5 months in the nab-paclitaxel–gemcitabine group as compared with 6.7 months in the gemcitabine group (*p* < 0.001) [9].

In a phase 2/3 randomised trial in patients with treatment-naïve metastatic pancreatic cancer with good ECOG PS 0–1, the combination of 5-FU, folinic acid, irinotecan and oxaliplatin (FOLFIRINOX) resulted in a better survival rate, but increased toxicity over gemcitabine alone; median overall survival 11.1 months versus 6.8 months respectively, *p* < 0.001 [10]. However, to date there are no identified predictive biomarkers to assess response to treatment for pancreatic cancer.

Several combination therapies with different cytotoxic agents have failed to show any clinical benefit in patients with advanced pancreatic cancer [11,12,13,14,15,16,17,18]. As a result of this unmet clinical need, several studies have been conducted with cytotoxic drugs and novel agents to identify an effective agent combination to control this aggressive disease. Pre-clinical evidence supports epidermal growth factor receptor (EGFR) involvement in the biology of pancreatic cancer [19,20]. Overexpression of EGFR type 1 (ErbB1/HER1) occurs in >90% of pancreatic cancer and is associated with a poorer prognosis [21].

A double-blind randomised Phase 3 trial conducted by the National Cancer Institute of Canada Clinical trials group (NCIC-CTG), comparing the gemcitabine/erlotinib combination with gemcitabine/placebo, demonstrated that the gemcitabine/erlotinib combination significantly improved progression-free survival (hazard ratio (HR) 0.77, 95% CI 0.64–0.92, *p* = 0.004) and overall survival (HR 0.82, 95% CI 0.69–0.99, *p* = 0.038)^.^ Median survival times were 6.24 months for the gemcitabine/erlotinib arm, versus 5.9 months for the gemcitabine/placebo arm with a one-year survival rate of 23% (95% CI 18%–28%) and 17% (95% CI 12%–21%) respectively [22]. As a result of this study, the Food and Drug Administration (FDA) approved the use of erlotinib in combination with gemcitabine for the first-line treatment of patients with locally advanced and metastatic pancreatic carcinoma [22].

The epidermal growth factor receptor is a transmembrane tyrosine kinase receptor that plays a major role in regulating cell proliferation and cell death [23,24]. It is comprised of four proteins: EGFR (HER1/ErbB1), ErbB2(HER2), ErbB3(HER3), ErbB4(HER4). Three pathways have been identified mediating the downstream effects of EGFR. The first pathway is RAS–RAF–mitogen-activated protein kinase (MAPK), where phosphorylated EGFR activates RAS and subsequently the MAP kinase pathway to affect cell proliferation, tumour invasion and metastasis. The second pathway is phosphatidylinositol-4,5-bisphosphate 3-kinase (PI3K)/AKT, which activates major cellular survival and anti-apoptosis signals, and the third pathway is the Janus kinases/signal transducers and activators of transcription (JAK/STAT) pathway, which activates transcription of genes associated with cell survival. Anti-EGFR monoclonal antibodies like cetuximab and panitumumab block ligand-induced receptor activation, while small molecule EGFR inhibitors such as erlotinib, gefitinib and lapatinib compete with adenosine triphosphate (ATP) to bind the catalytic domain of the kinase, which in turn inhibits EGFR autophosphorylation and downstream signalling [24]. The majority of targeted therapies against EGFR have not demonstrated the benefit that would have been theoretically expected in clinical trials in patients with advanced pancreatic cancer. Therefore, the benefit of adding EGFR-targeted agents to chemotherapy in the advanced setting is unclear.

This systematic review and meta-analysis was conducted to evaluate the efficacy and safety of addition of EGFR-targeted therapy to chemotherapy in patients with locally advanced and metastatic pancreatic cancer.

## 2. Results

**Figure 1:**
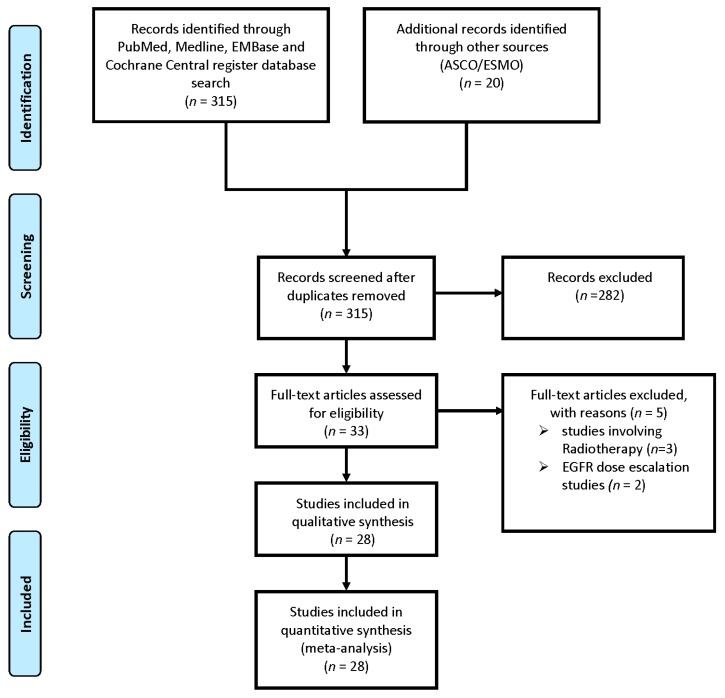
Flow chart outlining search strategy and details on final included and excluded studies in the meta-analysis. *n*: number; ASCO: American Society of Clinical Oncology; ESMO: European Society of Medical Oncology; EGFR: epidermal growth factor receptor.

A total of 3718 patients from 28 studies, including 7 randomised-controlled trials (RCTs) and 21 cohort studies (sample size ranging from 20 to 743), were included in this meta-analysis [25,26,27,28,29,30,31,32,33,34,35,36,37,38,39,40,41,42,43,44,45,46,47,48,49,50,51,52,53] (Figure 1). Ten studies were excluded from the final analysis. Amongst these, five were adjuvant studies, three studies involved radiotherapy, one was a retrospective study and one study involved dose escalation of erlotinib. Four studies reported on *KRAS* mutation status [32,40,46,53]. 

The majority of patients (62%) presented with metastatic disease. A total of 14% had locally advanced disease and in 24% of patients, disease stage was not available. Median age was 63 years (range 57–64), and 2065 patients (54%) were male. Sixty-eight percent of patients had an ECOG PS of 0–1. The primary endpoint of studies varied: progression-free survival (five studies), overall survival (nine studies), overall response rate (eight studies), maximum tolerated dose (one study), time to treatment failure (one study), safety (one study) and disease control rate (one study). The primary endpoint was not reported clearly in two studies. Ten treatment-related deaths were recorded in the twenty-eight studies. The reason for treatment discontinuation was not recorded in fourteen studies. A detailed description of selected studies included is provided in Table S1 (see Supplementary Material).

**Figure 2:**
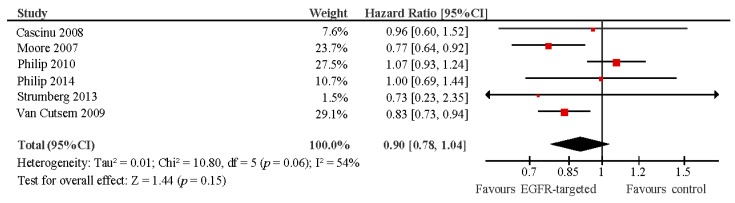
Forest plot showing hazard ratio for progression-free survival for addition of EGFR-targeted treatment to chemotherapy versus control. Experimental arm: EGFR-targeted therapy + chemotherapy; Control: chemotherapy; CI: confidence interval.

**Figure 3:**
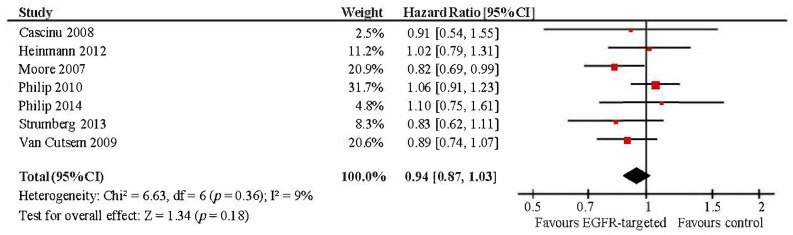
Forest plot showing hazard ratio for overall survival for addition of EGFR-targeted treatment to chemotherapy versus control. Experimental arm: EGFR-targeted therapy + chemotherapy; Control: chemotherapy; CI: confidence interval.

The addition of EGFR inhibitors to standard treatment did not improve progression-free survival (pooled HR 0.90, 95% CI 0.78–1.04, *p* = 0.15) (Figure 2) or overall survival (pooled HR 0.94, 95% CI 0.87–1.03, *p* = 0.18) (Figure 3). There was no association between grade (G)3/4 rash and overall survival (*R* = 0.03, *p* = 0.43).

**Figure 4:**
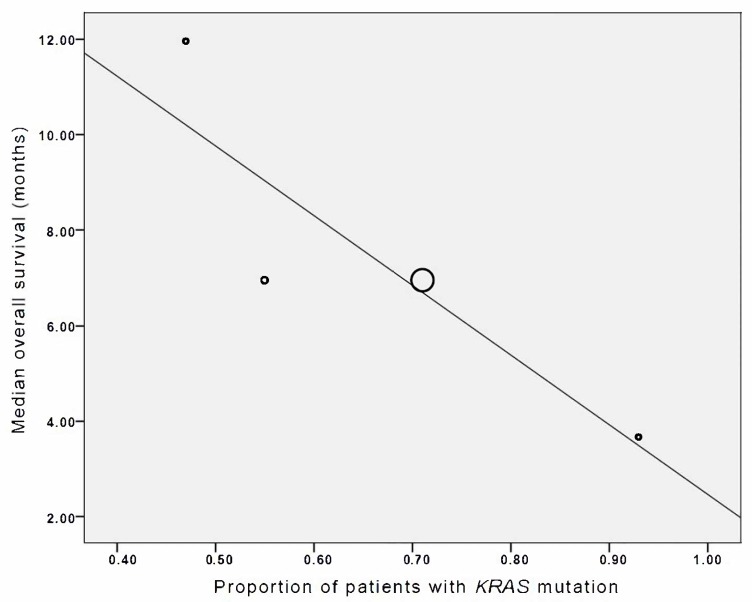
Individual study association of proportion of patients with *KRAS* mutations and median overall survival (in months). Each study is represented by a circle, and the area of the circle is proportional to the number of patients enrolled in each study. The gradient of the line represents the results of the meta-regression (*R* = −0.88).

Patients with *KRAS* mutations (*N* = 181 [68%]) derived less survival benefit from EGFR-targeted therapy to those without (*N* = 86) (*R* = −0.88, *p* < 0.001). Four studies [32,40,46,53] involving patients with *KRAS* mutations were included in this meta-analysis. (Figure 4). Survival benefit from EGFR-targeted therapy appeared greater among patients with locally advanced rather than metastatic disease (*R* = −0.69, *p* < 0.001).

**Figure 5:**
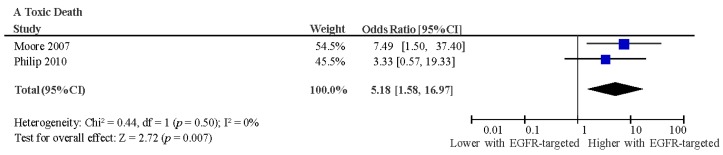

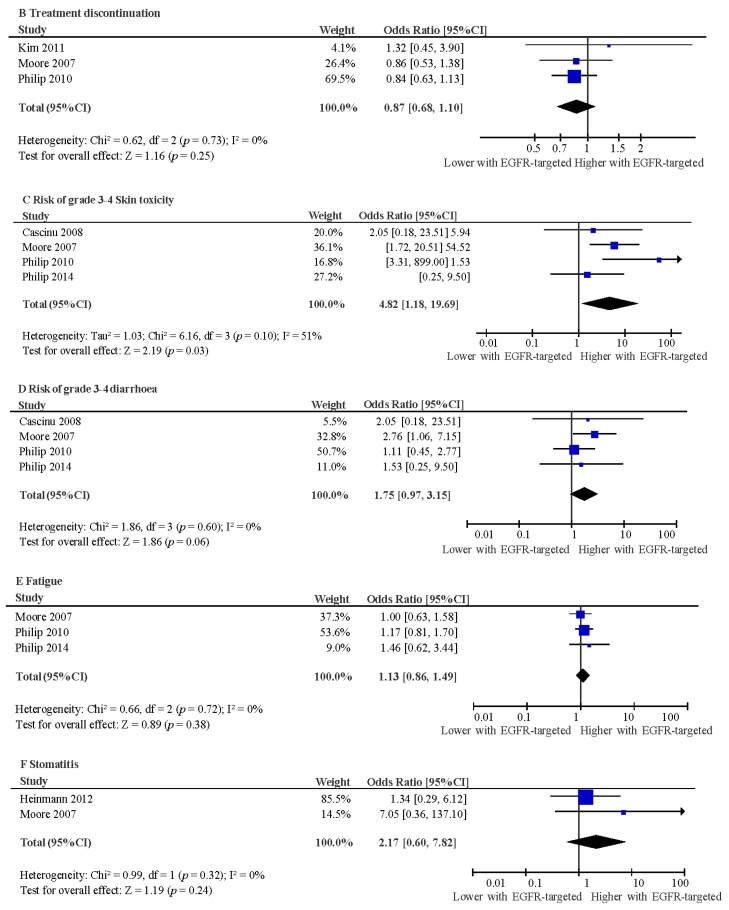
Forest plots showing odds ratio for toxic death (**A**); treatment discontinuation (**B**); risk of grade 3–4 skin toxicity (**C**); risk of grade 3–4 diarrhoea (**D**); fatigue (**E**) and stomatitis (**F**) for addition of EGFR-targeted treatment to chemotherapy versus control. Experimental arm: EGFR-targeted therapy + chemotherapy; Control: chemotherapy; CI: confidence interval.

There was significantly greater survival among studies with a higher proportion of male patients (*p* = 0.02), although this association was of very small magnitude (*R* = 0.092). There was no effect on survival of the proportion of patients included in studies with ECOG PS 0 or 1 (*p* = 0.65). This meta-analysis did demonstrate that EGFR-targeted therapy was associated with an increased risk of treatment-related death (pooled odds ratio (OR) 5.18, 95% CI 1.58–16.97 *p* = 0.0007) (10 treatment-related deaths out of 3718 patients included in meta-analysis) and toxicities including grade 3–4 rash (OR 4.82 95% CI 1.18–19.69 *p* = 0.03) and a near significant increase in grade 3–4 diarrhoea (OR 1.75, 95% CI 0.97–3.15, *p* = 0.25). There was no difference in treatment-related stomatitis (OR 2.17, 95% CI 0.60–7.82, *p* = 0.24) or fatigue (OR 1.13, 95% CI 0.86–1.49, *p* = 0.38). Additionally, there was no effect on treatment discontinuation without progression (OR 0.87, 95% CI 0.68–1.10, *p* = 0.25) (Figure 5).

## 3. Discussion

Pancreatic cancer is a disease with very poor prognosis. Many studies involving chemotherapy alone or in combination with novel agents have failed to demonstrate a significant impact on progression-free or overall survival in patients with locally advanced or metastatic pancreatic cancer [54,55,56,57].

This meta-analysis demonstrated that addition of EGFR inhibitors to chemotherapy increased the risk of severe toxicity and risk of treatment-related death (although this was numerically small), with an increased incidence of grade 3/4 skin rash and diarrhoea observed. It has previously been reported that patients treated with EGFR-targeted therapies commonly develop skin toxicities. Papulopustular rash and dry skin are the most commonly reported dermatological toxicities [58,59,60], which develop usually on the face, scalp, neck and upper trunk. The median time of onset is typically within 1–2 weeks from the start of therapy [58]. An association between the development of skin rash and efficacy has been explored in several studies using EGFR-targeted therapies. For example, studies conducted in different disease sites such as lung, head and neck, colorectal and pancreatic cancers have reported an association between increased skin toxicity and response rate, progression-free and overall survival [58,61,62,63,64]. A phase 2 study of cetuximab in combination with gemcitabine for the treatment of patients with advanced pancreatic cancer, demonstrated that the development of a grade 3 acneiform rash was associated with prolonged survival [53]. Wacker et al also analysed two randomised phase 3 studies, NCIC CTG BR. 21 (erlotinib versus placebo in patients with non-small cell lung carcinoma) and the NCIC CTG PA. 3 study (gemcitabine and erlotinib versus gemcitabine and placebo in patients with advanced pancreatic cancer), and concluded that the development of skin rash may be associated with increased response rate and that the presence of rash strongly correlated with overall survival in both studies [65,66]. However, this meta-analysis did not establish a link between the development of skin rash and improved progression-free or overall survival in a larger cohort of studies.

Numerous studies have previously reported that 70%–80% of patients with pancreatic adenocarcinoma carry an activating *KRAS* mutation [67], but the most recent data indicate that mutationally-activated *KRAS* is present in >90% of patients with pancreatic ductal adenocarcinoma [68,69,70,71], and these discrepancies may be due to variations in the method of *KRAS* analysis in the different studies. Previous studies in mouse models have demonstrated that KRAS is capable of initiating pancreatic ductal adenocarcinoma and continuous signalling is required for its progression and maintenance at the primary and metastatic sites [72,73].

Many retrospective studies in colon and lung cancers have demonstrated poor clinical outcomes as a result of treatment with EGFR tyrosine kinase inhibitors in patients harbouring *KRAS* mutations [74,75,76,77,78]. In contrast, a molecular subgroup analysis of the NCIC-CTG PA.3 study, Da Cunha et al failed to identify the EGFR gene copy number (GCN) and *KRAS* mutations as predictive markers of survival benefit [67]. In this meta-analysis, four studies reported on KRAS status, and these patients had a lower magnitude of survival benefit from the addition of EGFR-targeted therapy [32,40,46,53].

The findings of this meta-analysis also concluded that the addition of EGFR inhibitors to chemotherapy does not improve efficacy (survival) in an unselected patient population. However, patients with locally advanced pancreas cancer appeared to derive more survival benefit from EGFR-targeted therapy than those with metastatic disease. This is perhaps attributable to lower hypovascularity within the locally advanced tumours or because an altered tumour microenvironment may result in more effective drug delivery, although this is speculative only. Microscopically, pancreatic ductal adenocarcinoma cells form infiltrating gland-forming structures separated from each other by desmoplastic reaction. The non-neoplastic desmoplastic (stromal component) comprises more than 70% of the tumour mass and is commonly referred to as the tumour microenvironment. The stroma is very heterogeneous, consists of an extra cellular matrix and cells like inflammatory cells, pancreatic stellate cells, endothelial cells, fibroblasts and myofibroblasts. Hypovascularity and poor perfusion of the stroma creates a barrier to effective drug delivery and may be more evident in those with metastatic disease [79,80,81].

The EGFR and associated ligands are known to play an important role in tumorigenesis and these are expressed in the majority of solid malignancies [82,83,84]. However, the role of EGFRs in altering the tumour microenvironment has yet to be proven.

In addition, pancreatic ductal adenocarcinoma is an extremely heterogenous disease with three distinctive subtypes [85]. These subtypes of pancreatic ductal adenocarcinoma were reported as “classical” (representing 41.2% of analysed pancreatic cancers), “quasi-mesenchymal” (36.5%) and “exocrine-like” (22.3%) [85]. The classical type was found to be dependent strongly on *KRAS* signalling [85,86]. Collison et al. assessed the possibility that pancreatic ductal adenocarcinoma subtypes have subtype-specific drug responses by measuring responses to gemcitabine and erlotinib in human pancreatic ductal adenocarcinoma cell lines and reported that erlotinib was more effective in the classical-type cell lines [85,86,87]. It may be that those patients presenting with locally advanced rather than metastatic pancreas cancer exhibit more classical-type features, and thus have a better response to EGFR-targeted therapies, but this hypothesis has not been tested previously.

Limitations of this study are the inclusion of studies using various different EGFR-targeted therapies with differing administration schedules up to December 2014. There is the potential for publication bias with unpublished studies being excluded from the analysis. However, efficacy and safety outcomes were relatively homogeneous; it is a relatively large and robust dataset and is therefore likely representative of EGFR-targeted agents analysed in clinical trials to date.

In summary, in unselected patients with locally advanced or metastatic pancreatic cancer, the addition of EGFR-based therapy to chemotherapy increases toxicity, but does not improve efficacy. Further study of EGFR-based therapy in patients’ subgroups, either selected by clinical parameters (e.g., with locally advanced disease) or defined by molecular subtype (e.g., *KRAS* wild-type pancreatic cancer) may be warranted, and an increased understanding of primary resistance, role of intracellular redundancy and cross-talk amongst signalling pathways, acquired resistance, interaction of EGFR inhibitors with chemotherapy and potential biomarkers of their activity are necessary for successful trial design in this disease group.

## 4. Methods

### 4.1. Data Sources and Searches

This analysis was conducted in line with the Preferred Reporting Items for Systematic Reviews and Meta-Analyses guidelines [88]. An electronic literature search was carried out using MEDLINE (PubMed Ovid) EMBase and the Cochrane Central register of controlled trials up to December 2014. American Society of Clinical Oncology abstracts from 2012 to 2014 and European Society of Medical Oncology abstracts from 2012 to 2014 were also reviewed (it was expected that data presented earlier would be captured in full publications). Words such as “pancreatic cancer” or “locally advanced” or “EGFR-targeted therapy’’ or “erlotinib” or “gemcitabine” were included in the search.

### 4.2. Study Selection

Eligible studies included phase 2 (including combined phase 1/2 trials) and phase 3 studies examining the benefit of EGFR-targeted therapy in addition to chemotherapy in patients with locally advanced or metastatic pancreatic cancer. Included trials compared chemotherapy with epidermal growth factor receptor inhibitors to chemotherapy alone in patients with locally advanced or metastatic pancreatic cancer. Studies need to report a hazard ratio and 95% confidence interval or a *p*-value for overall survival or progression-free survival. Adjuvant and retrospective studies and those involving radiotherapy or dose-escalation were excluded. Duplicate publications were also excluded as were those not published in the English language. Two reviewers (Alison C. Backen and Mairéad G. McNamara) independently evaluated all of the titles identified by the search strategy. The results were then pooled, and all potentially relevant publications were retrieved in full. The same two reviewers then assessed the full articles for eligibility. Disagreement was resolved by consensus.

### 4.3. Data Extraction

From all the eligible trials, data were extracted on total number of participants, number of lines of previous treatment, age, ECOG performance status, *KRAS* mutation status, dosage of standard of care treatment and EGFR-targeted therapy, number of treatment-related deaths, treatment discontinuation and severity of toxicity as per the criteria used in each individual study. Hazard ratios were extracted preferentially from multivariable analyses, where available. Otherwise, hazard ratios from univariate analyses were extracted.

### 4.4. Statistical Analysis

Extracted data were combined into a meta-analysis using RevMan 5.3 analysis software (Cochrane Collaboration, Copenhagen, Denmark). Hazard ratios and their respective 95% CI were weighted and pooled using generic inverse variance [89]. Heterogeneity was assessed using the Cochran Q and *I*^2^ statistics. Fixed effect models were used if there was no evidence of statistical heterogeneity (Cochran Q *p* > 0.10 and *I*^2^ > 50%). Otherwise random-effects modelling was used. For safety and tolerability outcomes, odds ratios were calculated and pooled using Peto one-step method for toxic death and by the Mantel–Haenszel method for other toxicities. Meta-regression was used to explore factors associated with improved prognosis (all studies) and increased benefit from EGFR-targeted therapy (randomised controlled trials). Meta-regression comprised linear regression weighted by individual study sample size exploring the influence of proportion of patients with locally advanced presentation, ECOG 0 or 1, grade 3/4 diarrhoea and grade 3/4 rash on median survival (prognosis) and on the log of the hazard ratio for EGFR-targeted therapy. All statistical tests were two-sided, and statistical significance was defined as a *p* less than 0.05. No adjustment was made for multiple significance testing.

## Supplementary Materials

Supplementary materials can be found at www.mdpi.com/1422-0067/18/5/909/s1.
