# Supplementary material for: Targeting the Epidermal Growth Factor Receptor in Addition to Chemotherapy in Patients with Advanced Pancreatic Cancer: A Systematic Review and Meta-Analysis
Source: Int J Mol Sci. 2017 Apr 26;18(5):909. doi: 10.3390/ijms18050909 (PMC5454822; doi:10.3390/ijms18050909)
Supplement: Supplementary file 1 [file ijms-18-00909-s001.pdf]

Supplementary Materials: Targeting the Epidermal Growth Factor Receptor in Addition to Chemotherapy in Patients with Advanced Pancreatic Cancer: A Systematic Review and Meta-Analysis

Jaseela Chiramel, Alison C. Backen, Rille Pihlak, Angela Lamarca, Melissa Frizziero, Noor-ul-Ain Tariq, Richard A. Hubner, Juan W. Valle, Eitan Amir and Mairéad G. McNamara

Table S1. Characteristics of the studies included in the systematic review and meta-analysis.

Study	Design	N	Median Age	ECOG Performance Status	Presentation	Treatment Line	Grade ¾ A/E (N)	Treatment Regimen
Bengala et al. 2009 [25]	Phase 1/2	20	64	NR	LA = 4<br>M = 16	1st	5	Phase II trial of erlotinib in combination with increasing dose of gemcitabine given as fixed dose rate infusion in advanced pancreatic cancer (advanced pancreatic cancer); Gemcitabine 1500 mg/m^2 with an escalation of 500 mg/m^2 per cohort. Day 1 and 14 of a 28-day cycle , 6–8 courses. Erlotinib 100 mg/day until progression.
Kim et al. 2011 [26]	Phase 2	93	61	0–1	LA = 0<br>M = 93	1st	49	Randomised phase II trial of panitumumab, erlotinib, and gemcitabine versus erlotinib-gemcitabine (GE) in patients with untreated, metastatic pancreatic adenocarcinoma. Panitumumab 4 mg/kg every two weeks, Erlotinib 100 mg daily, Gemcitabine 1000 mg/m^2 weekly on a 28-day cycle.
Millela et al. 2010 [27]	Phase 2	46	64	NR	LA = 4<br>M = 41	1st	43	Phase II study of Erlotinib combined with fixed dose-rate gemcitabine (FDR-Gem) as first-line treatment for advanced adenocarcinoma of the pancreas. FDR-Gem was given weekly (1000 mg/m^2 at 10 mg/m^2/min); Erlotinib was given orally at 100 mg/day, continuously.
Modiano et al. 2012 [28]	NR	109	NR	NR	NR	NR	NR	Apricot-P: A randomised placebo-controlled phase II study of COX-2 inhibitor apricoxib or placebo in combination with Gemcitabine and Erlotinib in advanced or metastatic adenocarcinoma of the pancreas. Erlotinib 100 mg orally daily and apricoxib/placebo 400 mg orally daily and Gemcitabine.

Munoz Llarena et al. 2011 [29]	Phase 2	62	63	0–2	LA = 16<br>M = 46	1st	22	Gemcitabine (G) fixed-dose-rate infusion (FDR) plus Erlotinib (E) in patients with advanced pancreatic cancer (APC). G 1500 mg/m² was given by 150 min infusion (10 mg/m²/min) on days 1, 8, and 15 every 28 days combined with Erlotinib 100 mg/day orally.
Aranda et al. 2012 [30]	NR	153	63	NR	LA = 28<br>M = 125	1st	NR	Phase II open-label study of erlotinib in combination with Gemcitabine in unresectable and/or metastatic adenocarcinoma of the pancreas: relationship between skin rash and survival (Pantar study). Gemcitabine (1000 mg/m²/week, 3 weeks out of every 4 weeks) plus Erlotinib (100 mg/day orally continuously).
Ardavanis et al. 2009 [31]	Phase 2	27	63	NR	LA = 17<br>M = 10	1st	12	Biweekly Gemcitabine (GEM) in Combination with Erlotinib (ERL): An Active and Convenient Regimen for Advanced Pancreatic Cancer. GEM (2g/m² IV every two weeks) and 100 mg ERL orally every day, for at least 12 consecutive courses (6 cycles).
Cascinu et al. 2008 [32]	Phase 2	84	63	0–2	LA = 33<br>M = 61	1st	33	Cetuximab plus gemcitabine and cisplatin compared with gemcitabine and cisplatin alone in patients with advanced pancreatic cancer: a randomised, multicentre, phase II trial (SPaCe-01). Gemcitabine1000 mg/ m² + 35 mg/ m² cisplatin on days 1 and 8 of a 21-day cycle +/−cetuximab (250 mg/ m² weekly, after a loading dose of 400 mg/m²).
El-Rayes et al. 2011 [33]	Phase 2	20	57.9	0–1	LA = 4<br>M = 16	1st	21	A phase II study of isoflavones, erlotinib, and gemcitabine in advanced pancreatic cancer. Gemcitabine 1000 mg/m² on days 1, 8, and 15, and Erlotinib 150 mg once daily orally on day 1 to day 28. Soy isoflavones (Novasoy®) were administered at a dose of 531 mg twice daily orally starting day-7 until the end of study participation.
Feliu et al. 2011 [34]	Phase 2	42	62	0–2	LA = 6<br>M = 36	1st	28	Phase II study of a fixed dose-rate infusion of Gemcitabine in combination with Erlotinib in advanced pancreatic cancer. Gemcitabine 1200 mg/m² in 120-min infusion on days 1, 8 and 15, plus Erlotinib 100 mg orally once daily. Cycles were repeated every 28 days.

Heinmann et al. 2012 [35]	Phase 3	274	64	NR	LA = 43<br>M = 231	1st/2nd	NR	Gemcitabine plus erlotinib followed by Capecitabine versus Capecitabine plus Erlotinib followed by Gemcitabine in advanced pancreatic cancer: final results of a randomised phase 3 trial of the 'Arbeitsgemeinschaft Internistische Onkologie' (AIO-PK0104). Gemcitabine (1000 mg/m^2 intravenous weekly x 7, followed by 1-week rest, then weekly x 3, every 4 weeks, in combination with Erlotinib (150 mg daily) or oral Capecitabine (1000 mg/m^2 twice daily for 2 weeks, followed by 1-week rest) and Erlotinib (150 mg daily).
Hwang et al. 2012 [36]	Phase 2	22	63	0–1	LA = 3<br>M = 41	1st	22	A phase II trial of Erlotinib in combination with Gemcitabine and Cisplatin in advanced pancreatic cancer. Erlotinib 100 mg daily, 1000 mg/m^2 of Gem and 25 mg/m^2 of Cisplatin administered on days 1 and 8, respectively, every 3 weeks.
Ko et al. 2010 [37]	Phase 2	36	60	0–	LA = 0<br>M = 36	2nd-4th	36	A phase II study of Bevacizumab plus Erlotinib for Gemcitabine-refractory metastatic pancreatic cancer. Bevacizumab15 mg/kg every 21 days plus Erlotinib 150 mg daily.
Kulke et al. 2007 [38]	Phase 2	30	60	0–1	LA = 0<br>M = 30	2nd	20	Capecitabine plus Erlotinib in Gemcitabine-refractory advanced pancreatic cancer. Capecitabine1000 mg/m^2 twice daily for 2 weeks, followed by a 1-week break + Erlotinib 150 mg daily.
Kullmann et al. 2009 [39,40]	Phase 2	62	64.5	0–3	LA = 0<br>M = 32	1st	61	Cetuximab plus Gemcitabine/Oxaliplatin (GEMOXCET) in first-line metastatic pancreatic cancer: a multicentre phase II study. Cetuximab 400 mg/m^2 at first infusion followed by weekly 250 mg/m^2 combined with Gem 1000 mg/m^2 as a 100-min infusion on day 1 and Oxaliplatin 100 mg/m^2 as a 2-h infusion on day 2 every 2 weeks.
Lopez et al. 2013 [41]	Phase 2	32	NR	NR	LA = 0<br>M = 62	1st	4	Phase II trial of Erlotinib plus Capecitabine as first-line treatment for metastatic pancreatic cancer (XELTA study). Oral Capecitabine at 1,000 mg/m^2 twice daily on days 1-14, of a 21-day treatment cycle; and oral Erlotinib at 150 mg daily.
Moore et al. 2007 [42]	Phase 3	569	63.9	0–2	LA = 0<br>M = 32	1st	175	Erlotinib Plus Gemcitabine Compared with Gemcitabine alone in patients with advanced pancreatic cancer: A Phase III Trial of the National Cancer Institute of Canada Clinical Trials Group. Gemcitabine (1000 mg/m^2) was given IV on days 1, 8, 15, 22, 29, 36 and 43 followed by a one-week rest in cycle 1, and on days 1, 8 and 15 in all subsequent four week cycles. Erlotinib or placebo was taken orally at 100 mg/day or 150 mg/day.

Oh et al. 2012 [43]	Phase 2	47	57.3	0–3	LA = 138<br>M = 431	1st	13	A phase II trial of Erlotinib in combination with Gemcitabine and Capecitabine in previously untreated metastatic/recurrent pancreatic cancer: combined analysis with translational research. Erlotinib was given at a dose of 100 mg daily from Day 1 to Day 28. 1000 mg/m^2 of Gemcitabine was given on Day 1,8,15 and 1660 mg/m^2/day of Capecitabine was given from Day 1 to 21, repeated every week.
Okusaka et al. 2010 [44]	Phase 2	106	62	0–3	LA = 0<br>M = 47	1st	175	Phase II study of Erlotinib plus Gemcitabine in Japanese patients with unresectable pancreatic cancer. Erolotinib 100 mg/day orally + Gemcitabine (1000 mg/m^2 intravenously) on days 1, 8 and 15 of a 28-day cycle.
Park et al. 2013 [45]	Phase 2	69	62	0–1	LA = 18<br>M = 88	1st	21	Phase II Trial of Erlotinib plus Gemcitabine chemotherapy in Korean patients with advanced pancreatic cancer and prognostic factors for chemotherapeutic response. Erlotinib (100 mg) daily, and Gemcitabine 1000 mg/m^2 as infused intravenously over 30 min weekly (days 1, 8, and 15) followed by a 1-week rest per 4 weeks cycle.
Phillip et al. 2010 [46]	Phase 3	743	64	0–1	LA = 6<br>M = 63	1st	495	Phase III Study comparing Gemcitabine plus Cetuximab versus Gemcitabine in patients with advanced pancreatic adenocarcinoma: Southwest Oncology Group–Directed Intergroup Trial S0205. Gemcitabine (intravenously, 1000 mg/m^2) weekly for 7 weeks, followed by 1 week off, then weekly for 3 out of 4 weeks thereafter. Cetuximab (intravenously at a loading dose of 400 mg/m^2 on week 1, followed by weekly maintenance doses of 250 mg/m^2.
Philip et al. 2014 [47]	Phase 1b/2	116	63	0–2	LA = 160<br>M = 583	1st	NR	Dual blockade of Epidermal Growth Factor Receptor and Insulin-Like Growth Factor Receptor–1 Signaling in metastatic pancreatic cancer Phase Ib and Randomised Phase II Trial of Gemcitabine, Erlotinib, and Cixutumumab versus Gemcitabine plus Erlotinib (SWOG S0727). Gem 1000 mg/m^2 intravenously once weekly for 3 of 4 weeks. Erlotinib 100 mg orally, daily, continuously. Phase 1b-Cixutumumab 6 mg/kg (starting dose level) was administered on days 1, 8, 15, and 22 of each 28-day cycle in addition to the Gem and Erlotinib. The starting dose of cixutumumab (6 mg/kg) in the combination was used in the randomised phase II portion of the study.

Renouf et al. 2014 [48]	Phase 2	49	62	0–2	LA = 0<br>M = 116	2nd	21	A phase II study of erlotinib in Gemcitabine-refractory advanced pancreatic cancer. Erlotinib was given at an initial dose of 150 mg/day, and the dose was escalated by 50 mg every 2 weeks (to a maximum of 300 mg/day) until >grade 1 rash or other dose limiting toxicities occurred.
Safran et al. 2011 [49]	Phase 2	29	64	0–	LA = 0<br>M = 29	1st	31	Lapatinib and Gemcitabine for metastatic pancreatic cancer. Gemcitabine 1000 mg/m² intravenously weekly for 3 weeks of a 4-week period, with Lapatinib 1500 mg/day orally.
Stumberg et al. 2013 [50]	Phase 2	186	63.6	0–2	NR	1st	0	Phase II, randomised, double-blind placebo-controlled trial of Nimotuzumab plus Gemcitabine compared with Gemcitabine alone in patients with advanced pancreatic cancer. Gemcitabine 1000 mg/m² intravenously once weekly (days 1, 8, 15; every 28 days) and nimotuzumab: fixed dose of 400 mg once weekly as a 30 min infusion, or placebo, until progression or unacceptable toxicity.
Van Custem et al. 2009 [51]	Phase 3	607	61.5	0–2	NR	1st	NR	Phase III Trial of Bevacizumab in combination with Gemcitabine and Erlotinib in patients with metastatic pancreatic cancer. Gemcitabine 1000 mg/m²/week, Erlotinib (100 mg/day), and Bevacizumab 5 mg/kg every 2 weeks or Gemcitabine, Erlotinib and placebo.
Watkins et al. 2014 [52]	Phase 1/2	44	63	0–1	LA = 22<br>M = 22	1st	86	The combination of a chemotherapy doublet (Gemcitabine and Capecitabine) with a biological doublet (Bev and Erlotinib) in patients with advanced pancreatic adenocarcinoma. Gemcitabine (1000 mg/m² Days 1, 8, 15), Capecitabine (1400 mg/m² Days 1–21), Erlotinib (100 mg daily) and Bevacizumab (5 mg/kg Days 1, 15) every 28 days.
Xiong et al. 2004 [53]	Phase 2	41	61.1	0–1	LA = 6<br>M = 35	1st	113	Cetuximab, a monoclonal antibody targeting the epidermal growth factor receptor, in combination with Gemcitabine for advanced pancreatic cancer: a multicenter phase II Trial. Cetuximab at an initial dose of 400 mg/m², followed by 250 mg/m² weekly for 7 weeks. Gemcitabine was administered at 1000 mg/m² for 7 weeks, followed by 1 week of rest. In subsequent cycles, Cetuximab was administered weekly, and Gemcitabine was administered weekly for 3 weeks every 4 weeks.
